# Emerging scientists in analytical sciences: Carla Kirschbaum

**DOI:** 10.1002/ansa.202200037

**Published:** 2022-10-21

**Authors:** Carla Kirschbaum

**Affiliations:** ^1^ Institute of Chemistry and Biochemistry Freie Universität Berlin Berlin Germany; ^2^ Department of Molecular Physics Fritz‐Haber‐Institut der Max‐Planck‐Gesellschaft Berlin Germany


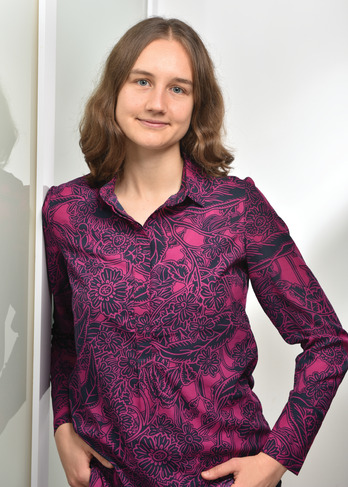
 Carla Kirschbaum is a fourth‐year PhD student in the group of Prof. Kevin Pagel at the Freie Universität Berlin and the Fritz Haber Institute of the Max Planck Society. She studied chemistry in Berlin and first joined the group in 2017 for her bachelor's thesis. After a study‐ and research stay in Paris, she returned to Berlin to complete her master's degree and then seamlessly continue with her doctorate. Since her bachelor's thesis, Carla has been exploring mass spectrometry‐based techniques for the structural analysis of lipids. She has received numerous grants and awards for her work and is actively involved in the Young Scientists interest group of the German Society for Mass Spectrometry (DGMS) to foster exchange among young researchers in the field of mass spectrometry.

1

**How did you get involved in the field of analytical sciences?**

In fact, my university does not have an analytical chemistry division, and therefore it was not until my bachelor's thesis that I got involved in the field of analytical sciences. During my undergraduate studies, we learned the basics of several analytical techniques in order to analyse substances that we synthesized in the organic chemistry lab courses; however, analytical chemistry was mainly regarded as an auxiliary technique for the synthetic chemist. In my second year, I attended the lecture on bioorganic chemistry by Prof. Kevin Pagel, which became my favourite course in my entire undergraduate studies. Afterwards, Kevin Pagel offered me to do my bachelor's thesis in his group where I got my first hands‐on experience with mass spectrometers. In his lab, which is partly located at the Fritz Haber Institute of the Max Planck Society, I used electrospray‐mass spectrometry hyphenated with ion mobility spectrometry to study isomeric phospholipids. Although the project did not yield the desired results, my interest in mass spectrometry and lipid analysis had been sparked. In the following year, when I moved to Paris for my master's studies, I continued to work in the field of mass spectrometry. As part of a research internship, I got to know other types of mass spectrometers and ionization techniques at the mass spectrometry platform of Sorbonne Université. In the lab of Prof. Sandrine Sagan, I studied the interactions between cell‐penetrating peptides and membrane lipids by crosslinking mass spectrometry.^1^

2

**What is the topic of your PhD studies?**

After my return from Paris, I finished my master's studies in Berlin and rejoined the Pagel lab for my master's thesis, which laid the groundwork for my PhD thesis. During my master's thesis, I was trained on an instrument developed in the group of Prof. Gert von Helden at the Fritz Haber Institute which combines mass spectrometry and infrared ion spectroscopy. Gas‐phase infrared spectra of mass‐to‐charge‐selected ions are obtained by encapsulating the ions in superfluid helium droplets and monitoring the release efficiency from the droplet as a function of the infrared laser wavenumber. Superfluid helium droplets have an intrinsic temperature of 0.4 K in vacuum and are thus perfect cryostats to prevent ion heating and achieve very high spectral resolution. I used this technique for the analysis of lipids and found that isomeric glycolipids are unambiguously distinguishable based on their vibrational fingerprints. In my first PhD project, we showed that our instrument is capable of determining the isomeric composition of natural glycolipid extracts using minute sample quantities.^2^ Because gas‐phase infrared spectra of lipids were not reported in the literature at the beginning of my PhD, I could freely choose my projects within this unexplored area. Following the glycolipid project, I investigated other kinds of isomerism in other lipid classes, for example double bond regio‐ and stereoisomers in sphingolipids and fatty acids.^3^, ^4^ Towards the end of my PhD studies, I became interested in studying lipid fragmentation mechanisms in tandem mass spectrometry. Infrared spectroscopy in combination with density functional theory can be employed to determine the structure of lipid fragments and thereby confirm or disprove proposed fragmentation mechanisms.^5^, ^6^ Many projects emerged in collaboration with other research groups, including the group of Prof. Luc Teyton at the Scripps Research Institute, Prof. Christoph Arenz at Humboldt‐Universität zu Berlin and Prof. Stephen Blanksby at Queensland University of Technology. Overall, my PhD thesis is a collection of applications of cryogenic infrared ion spectroscopy for the structural identification of lipids and for gaining fundamental knowledge about their fragmentation in tandem mass spectrometry.


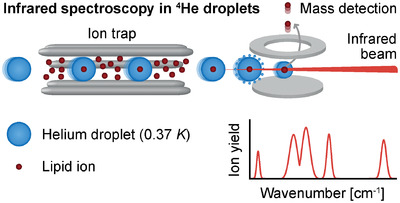


3

**What was your biggest achievement during this time?**

I find it difficult to name a single achievement as the most important one; however, I can highlight two challenges that I have successfully mastered during my PhD studies. During my first project on glycolipids, we had a hard time getting the paper published and were requested to prove that the technique works for biological samples. We obtained biological lipid extracts from the Scripps Research Institute; however, it was extremely difficult to clean the samples and separate the glycolipids from the highly abundant and easy‐to‐ionize phospholipids. It was a very tedious and frustrating work to establish the cleanup procedure while only having limited amount of sample at hand. In the end, we were able to detect the glycolipids we were looking for and measure infrared spectra of reasonable quality. That was the most rewarding day in my first year as a PhD student. The second achievement that I would like to point out has to do with the free‐electron laser that we use as a light source for our experiment. I am very fascinated with lasers and attended a course on laser technology at the university during my first PhD year. In my second year, I had the unique opportunity to be trained as a free‐electron laser operator together with my colleague Kim Greis over several months. We operated the laser for 3 weeks during the absence of the staff operators and, miraculously, the laser ran at peak performance throughout the entire 3 weeks. That was the most productive measurement time ever for our group and we are still relieved that we did not break anything.

4

**Can you describe current trends in analytical instrumentation for cryogenic infrared ion spectroscopy?**

The reason why cryogenic infrared ion spectroscopy is still a niche application, despite its immense potential, is that the instrumentation is complicated, sensitive and expensive. Therefore, only a few specialized laboratories worldwide currently develop and work with this technique. The main issues are the need for cryogenic cooling and a sufficiently powerful light source. In my estimation, cryogenic infrared spectroscopy in helium droplets will remain a technique for fundamental research. Although it provides the ultimate spectral resolution, the instrumentation for generating superfluid helium droplets plus the need for an in‐house free‐electron laser makes it virtually impossible to commercialize. However, considerable progress is currently being made in the development of tabletop infrared lasers, miniaturization of cryogenic cooling devices and general simplification of the experimental setup for cryogenic infrared ion spectroscopy. I think that especially tagging spectroscopy has the potential to be integrated into analytical workflows for the structural analysis of biomolecules. This technique involves the tagging of the analyte ion with a gas atom or molecule at cryogenic temperatures, which can be detached by the absorption of a single resonant infrared photon. Therefore, the technique works with tabletop lasers and combines compactness with high spectral resolution. Because infrared spectroscopy is orthogonal to mass spectrometry and can reveal the coexistence of isomers below a single mass peak, it is of utmost interest to get this technique into analytical laboratories. I think that in a few decades, infrared ion spectroscopy will be a standard application.

5

**What advice would you give to recent PhD starters?**

At the beginning of my PhD, I was applying for doctoral fellowships and was hence forced to define my planned projects and establish a working plan. This cost me quite some time and was difficult to do at the very beginning of my doctorate. However, in retrospect, it was one of the most useful activities during my first weeks of being a PhD student. I think that it is worth the effort to define goals and think thoroughly about potential projects rather than jumping into the doctorate without any plan. Of course, no plan ever works out but it is important to know the direction and have some structure to get back to. Also, I would advise all PhD starters to take advantage of the many learning opportunities that are offered specifically for doctoral students by many different institutions. I attended seminars at the Freie Universität Berlin, the Dahlem Research School and the Studienstiftung des deutschen Volkes; both specialized courses on chemistry and lasers, and more general courses about giving presentations, design principles, conflict management and many more. I even learned Spanish as a new foreign language, which I continue to practise with my Mexican colleague América Torres. The early PhD phase was a time when I enjoyed great freedom, whereas at a later stage of the PhD it becomes more difficult to find time to attend additional courses out of pure interest. I also find it important to attend conferences and other networking events to get in contact with other students and see what is being done in other research groups. My first conference was the annual Young Scientists meeting of the German Society for Mass Spectrometry, which I was later in charge of organizing. Such small conferences offer an excellent opportunity especially for PhD starters to present their work in a comfortable atmosphere.

6

**What will you do after your PhD study?**

I have to admit that I got caught by the analytical sciences and I want to continue working in this field. After finishing my PhD in a few months from now, I will join the group of Prof. Carol Robinson at the University of Oxford to work with mass spectrometry in a biochemical context. I will investigate how lipids influence the structure and function of membrane proteins in a complex biological environment. The ultimate goal is to combine native mass spectrometry with spectroscopy to determine the exact structure of lipids that modulate membrane proteins and to study the biological effects of structural changes in the lipids. For example, cancer cells can produce unusual lipids that might interact differently with membrane proteins and cause aberrant protein function.

7

**Where do you see yourself in 10 years?**

If nothing gets in my way, I still see myself working in analytical sciences in 10 years. I hope that I can combine all the different techniques that I have learned throughout my studies to investigate lipids in a biological context. I can imagine that in 10 years infrared ion spectroscopy is sufficiently mature to study not only isolated, purified lipids, but to investigate their interactions with other biomolecules on a functional biological level. In my PhD studies, I have established that cryogenic infrared spectroscopy is a highly diagnostic tool for lipid structural analysis and I would like to build on that knowledge to study more complex problems. Ideally, I see myself in an interdisciplinary environment with biologists, chemists and physicists. In the long term, I would like to return to Berlin or surroundings because both my and my husband's family and friends are located there and form an important part of our lives.

8

**Can you say something about your hobbies outside the laboratory?**

I enjoy all kinds of outdoor activities, especially if they involve walking, running, biking or skating. This summer, my university organized a running event across the campus, to which our whole group signed up. We had neon pink headbands made with our nerdy team name ‘hurried heavy helium droplets’ embroidered. All of us flew across the finish line like helium droplets and we had a nice get‐together afterwards. These are the kinds of activities that I enjoy very much. Apart from my colleagues, I like to spend time with my family and friends who do not want to talk about science all the time. I would like to take this opportunity to thank my family, friends and colleagues for their continued support.


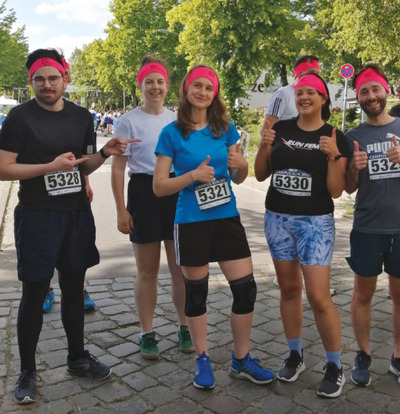


## CONFLICT OF INTEREST

The author declares no conflict of interest.

## References

[1] Bechtella L , Kirschbaum C , Cosset M , et al. Benzophenone photoreactivity in a lipid bilayer to probe peptide/membrane interactions: simple system, complex information. Anal Chem. 2019;91:9102‐9110. 31251038 10.1021/acs.analchem.9b01584

[2] Kirschbaum C , Greis K , Mucha E , et al. Unravelling the structural complexity of glycolipids with cryogenic infrared spectroscopy. Nat Commun. 2021;12:1201. 33619275 10.1038/s41467-021-21480-1 PMC7900115

[3] Kirschbaum C , Saied EM , Greis K , et al. Resolving sphingolipid isomers using cryogenic infrared spectroscopy. Angew Chem Int Ed. 2020;59:13638‐13642. 10.1002/anie.202002459 PMC7496694 32291895

[4] Kirschbaum C , Greis K , Lettow M , et al. Non‐covalent double bond sensors for gas‐phase infrared spectroscopy of unsaturated fatty acids. Anal Bioanal Chem. 2021;413:3643‐3653. 33956167 10.1007/s00216-021-03334-3 PMC8141490

[5] Kirschbaum C , Greis K , Polewski L , et al. Unveiling glycerolipid fragmentation by cryogenic infrared spectroscopy. J Am Chem Soc. 2021;143:14827‐14834. 34473927 10.1021/jacs.1c06944 PMC8447261

[6] Kirschbaum C , Greis K , Gewinner S , et al. Cryogenic infrared spectroscopy provides mechanistic insight into the fragmentation of phospholipid silver adducts. Anal Bioanal Chem. 2022;414:5275‐5285. 35147717 10.1007/s00216-022-03927-6 PMC9242943

